# Turning Fe-Rich Waste into an Advanced Electrocatalyst for the Production of H_2_ and Useful Carboxylates

**DOI:** 10.1021/acsomega.5c08418

**Published:** 2025-12-08

**Authors:** Karthik Eswaran, Rajini Murugesan, Arthanareeswari Maruthapillai, Anantharaj Sengeni

**Affiliations:** † Department of Chemistry, Faculty of Engineering and Technology, 93104 SRM Institute of Science and Technology, Kattankulathur 603203, Tamil Nadu, India; ‡ Laboratory for Electrocatalysis and Energy, Department of Chemistry, Indian Institute of Technology, Kanpur 208 016, Uttar Pradesh, India

## Abstract

In this study, discarded bike tire rim wires composed primarily of iron (Fe) were repurposed into electrocatalysts through a corrosion-assisted surface activation strategy. The wires were acid-treated, heat-cleaned, and then subjected to a 10-day corrosion process in a NaOH and Ca­(OCl)_2_ solution. Their catalytic activities toward the hydrogen evolution reaction (HER), oxygen evolution reaction (OER), and small-molecule oxidation (such as methanol, ethanol, and urea) were systematically evaluated. Optimal HER and OER performances were observed on the eighth and third days of activation, respectively, reflecting their distinct mechanistic pathways in alkaline media. Tafel and electrochemical impedance spectroscopy revealed that both intrinsic catalytic properties and surface area enhancements contributed to activity improvements. The repurposed Fe-rich electrodes exhibited high selectivity for the controlled oxidation of methanol and ethanol into carboxylic acids, which is attributed to their weak OH adsorption. Remarkably, urea oxidation occurred at significantly lower potentials, likely due to favorable Fe–NH_2_ interactions. Surface and compositional analyses confirmed that the wires were composed of 99.76% Fe, with trace amounts of Cr and Ni. This work highlights the potential of transforming metallic waste into functional electrocatalysts, offering a dual benefit of sustainable resource utilization and mechanistic insights for water electrolysis and wastewater valorization applications.

## Graphical abstract

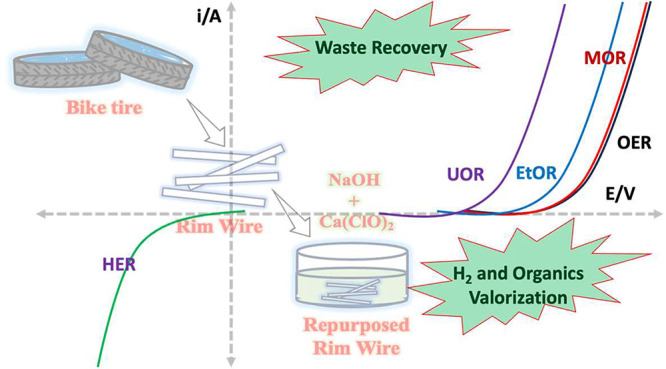

## Introduction

The drive toward sustainable energy systems has accelerated research into electrochemical processes capable of converting abundant molecules into clean fuels and valuable chemicals.^1–4^ Among these processes, electrolysis of water for hydrogen generation and the electrocatalytic oxidation of small organic molecules such as methanol, ethanol, and urea have gained prominence due to their central role in decarbonizing the energy landscape and closing the carbon cycle.^2,3,5^ Water electrolysis, in particular, represents a cornerstone technology for renewable hydrogen production, a critical feedstock for fuel cells and chemical synthesis.^5^ Meanwhile, direct electrooxidation of alcohols and urea offers an attractive strategy for fuel cells and wastewater valorization.^6–11^ However, the widespread adoption of these technologies is impeded by the reliance on scarce, high-cost noble metals (e.g., Pt, Ir, Ru) as electrocatalysts.^12–14^ Consequently, significant efforts have been dedicated to identifying alternative catalysts derived from earth-abundant metals, industrial byproducts, and waste resources to reduce economic and environmental barriers.^2,3^ For example, transition metal-based materials (e.g., Fe, Co, Ni) show better activity and stability in alkaline medium as they can make a favorable −OH bond, which initiates the water splitting reaction in alkaline solution.^15–17^ The reutilization of metallic wastes and discarded materials as electrocatalysts has emerged as a promising route to minimize the ecological footprint of catalyst fabrication.^18–20^ This approach is synergistic with the principles of green chemistry and circular economy, wherein waste materials are revalorized rather than disposed of. For example, scrap stainless steel,^21–25^ decommissioned battery components,^26–30^ and spent catalysts have been processed into functional electrodes after appropriate surface modification. Notably, iron-based materials (widely available in steel and iron alloys) have drawn considerable attention due to their low cost, chemical tunability, and decent catalytic performance for the oxygen evolution reaction (OER) and hydrogen evolution reaction (HER) in alkaline media.^31–34^ Iron oxides, hydroxides, and oxyhydroxides, in particular, are known to exhibit intrinsic OER activity, especially when combined with other 3d transition metals such as Ni and Co.^32,35^ Nonetheless, despite the broad availability of iron-rich waste, there remains a gap in methodologies that transform these resources into high-surface-area, catalytically active electrodes through mild and scalable treatments. Chemical oxidation is an effective strategy to engineer surface compositions and defect sites on metallic substrates, thereby tuning their electrochemical performance.^25,36–41^ Oxidative pretreatments can produce porous oxide shells, increase hydrophilicity, and expose high-energy facets or active defects that facilitate the charge transfer and adsorption of reaction intermediates. For instance, the use of hypochlorite-based oxidants has been reported to create roughened, oxide-rich layers on steel and iron electrodes, improving their catalytic activity toward OER.^25,40^ However, systematic studies connecting the duration of chemical oxidation, the resulting surface chemistry, and electrocatalytic performance across multiple reactions, including both water splitting and organic oxidation, are relatively scarce.

Parallel to advances in water electrolysis, the electrooxidation of small organics such as methanol, ethanol, and urea has attracted sustained interest for energy conversion and pollution mitigation.^3^ Direct methanol and ethanol fuel cells promise high energy densities and facile liquid fuel storage but are hindered by sluggish kinetics and incomplete oxidation pathways on conventional catalysts.^12^ Typically, noble metals such as Pt and Pd facilitate deep oxidation to CO_2_, yet their high cost and vulnerability to poisoning limit practicality.^42–45^ Iron-based materials often show partial oxidation pathways, yielding formate and acetate as primary products instead of CO_2_.^46–48^ While this incomplete oxidation is traditionally viewed as a limitation in fuel cell technology, it can be leveraged for selective synthesis of value-added chemicals if appropriately controlled. Indeed, recent studies have demonstrated that tuning surface properties and reaction conditions can achieve high selectivity for partial oxidation, highlighting the importance of developing inexpensive, selective catalysts derived from abundant resources.

In this context, we present an approach to repurpose the rim wires recovered from disposed bicycle tires, an iron-rich waste stream, as catalytic electrodes for a suite of electrochemical conversions, including HER, OER, methanol oxidation reaction (MOR), ethanol oxidation reaction (EtOR), and urea oxidation reaction (UOR).

These rim wires, primarily composed of iron (∼99.76%), were chemically modified using calcium hypochlorite (Ca­(OCl)_2_), a relatively benign oxidant, to engineer the surface oxide layers and enhance the catalytic activity. By systematically varying the oxidation time, we optimized the balance between surface passivation and active site generation, achieving appreciable HER and OER performance in an alkaline electrolyte. Notably, in MOR and EtOR, the modified rim wires demonstrated a remarkable preference for partial oxidation, producing formic and acetic acids without progressing to complete mineralization into CO_2_. This selective behavior offers opportunities for controlled organic transformations and coelectrolysis strategies, particularly in UOR, where low onset potentials further underscore the catalytic potential of the system. Overall, this work underscores how common waste materials, when judiciously modified, can serve as versatile electrocatalysts for both water splitting and selective organic oxidation (Scheme 1). By combining low-cost feedstocks with mild processing methods, we aim to contribute to a sustainable platform for decentralized hydrogen production and organic valorization, bridging the gap between resource recovery and clean energy technologies.

**Scheme 1:**
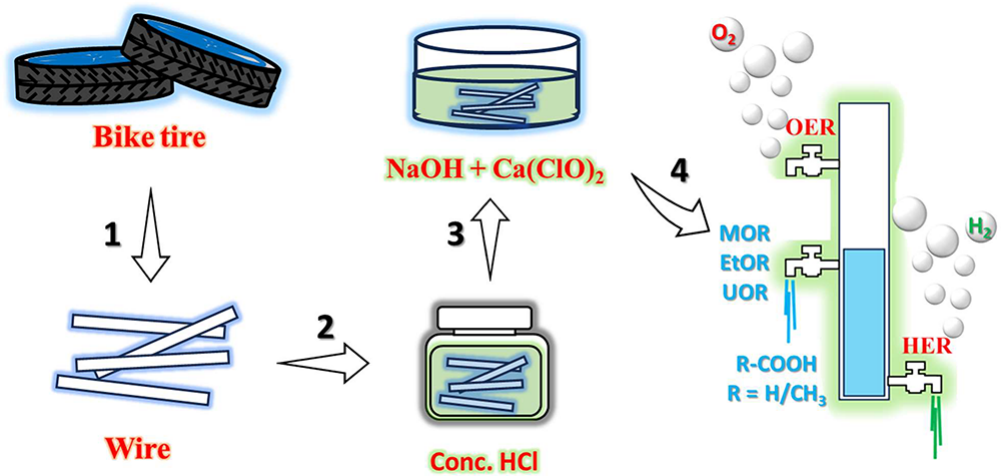
Step-by-Step Procedure Followed in Recovering and Repurposing the Rim Wire from Discarded Bike Tires to Be Used as a Catalytic Electrode for HER, OER, MOR, EtOR, and UOR

## Experimental Section

### Material Preparation and Pretreatment

#### Collection and Isolation of Rim Wire

Waste motorbike tires were sourced from local repair shops. The metallic rim wires were manually extracted from the disposed tires. The wires were mechanically cleaned to remove adherent rubber and debris. The diameter was measured using a calibrated vernier caliper, yielding a radius (*r*) of 0.0457 cm on average. For electrochemical experiments, each electrode segment was cut to a length of 3.5 cm, corresponding to an exposed geometric surface area calculated by the following cylinder formula:

$$A=2\pi rh=2\times 3.1416\times 0.0457\;\mathrm{cm}\times 3.5\;\mathrm{cm}=1.005\;{\mathrm{cm}}^{2}$$

#### Acid Cleaning

The cleaned wires were first heated to ∼120 °C in air to facilitate the removal of organic residues and partially oxidize the surface. Subsequently, the wires were immersed in 6 M hydrochloric acid (HCl) at ambient temperature for 3 h to dissolve residual scale, rust, and superficial oxides.

#### Selective Chromium Removal

To deplete chromium, which is known to impede catalytic activity in alkaline electrochemistry, the acid-cleaned wires were soaked in a solution comprising 3 M sodium hydroxide (NaOH) supplemented with 1 mg of commercial bleaching powder (calcium hypochlorite, Ca­(OCl)_2_). During soaking, the in situ generation of sodium hypochlorite (NaOHO) occurs via the reaction of NaOH with Ca­(OCl)_2_, which subsequently oxidizes chromium to chromyl chloride (CrO_2_Cl_2_), facilitating its leaching from the metallic surface.

#### Optimization of Pretreatment Duration

To identify the optimum treatment time, wires were soaked in the NaOH/Ca­(OCl)_2_ solution for different durations (1, 2, and 3 days). Extended soaking beyond 1 day led to visible corrosion and mechanical weakening without significant improvement in electrochemical performance. Therefore, a 24 h treatment was selected for all subsequent experiments.

##### Physicochemical Characterization

Powder X-ray diffraction (XRD) patterns of the as-recovered, acid-cleaned, and chemically oxidized wires were recorded on a diffractometer by using Cu Kα radiation (λ = 1.5406 Å). Data were collected in the 2θ range of 10–100°, with a step size of 0.02° and a scan rate of 3° min^–1^ to identify phase composition. Scanning electron microscopy (SEM) and energy-dispersive X-ray spectroscopy (EDS) analyses were also performed to learn the surface morphology and elemental distribution. SEM images were obtained at an accelerating voltage of 55 kV. EDS mapping confirmed the elemental composition and monitored the chromium removal. X-ray photoelectron spectroscopy (XPS) measurements were performed to examine the surface chemical states. Survey and high-resolution spectra for Fe 2p, Ni 2p, Cr 2p, and C 1s were acquired.

##### Electrochemical Characterization

Electrochemical measurements were performed in a standard three-electrode setup by using a potentiostat/galvanostat (OrgaFlex OGF500). The prepared rim wire served as the working electrode. A Hg/HgO reference electrode filled with 1.0 M KOH (MMO) was used as the reference electrode and a carbon cloth as the counter electrode. All potentials were converted to the reversible hydrogen electrode (RHE) scale, where applicable. Linear sweep voltammetry (LSV) was conducted at a scan rate of 5 mV s^–1^ in 1 M KOH (HER/OER baseline), 1 M KOH + 0.5 M MOR, 1 M KOH + 0.5 M EtOR, and 1 M KOH + 0.5 M urea (UOR). Onset potentials were determined by extrapolating the linear portion of the current–potential curve to the baseline. Among all of the electrolytes, urea oxidation exhibited the lowest onset overpotential. Electrochemical impedance spectroscopy (EIS) was performed at an overpotential of 1.624 V vs RHE, with frequencies ranging from 100 kHz to 0.1 Hz and a perturbation amplitude of 10 mV. Nyquist and Bode plots were generated to assess the charge-transfer resistance (*R*_ct_) and capacitive behavior. The catalytic stability was evaluated by applying a constant potential of 1.2 V vs RHE using chronoamperometry (CA) for 12 h in each electrolyte. The current retention and potential drift were recorded to assess the durability. Tafel slopes were derived by plotting the overpotential versus the logarithm of current density. Additionally, sampled current voltammetry (SCV) was employed to validate Tafel slope estimations by incrementally increasing the applied potential in 50 mV steps with equilibration periods.

##### Product Analysis of MOR and EtOR

Postelectrolysis liquid products of methanol and ethanol oxidation were analyzed by ^1^H and ^13^C NMR spectroscopy using deuterated water as a solvent. The spectra confirmed the selective formation of formic and acetic acids, with no significant signals corresponding to CO_2_-related species. The depletion of urea concentration after electrolysis was verified by comparing pre- and postelectrolysis IR spectra. Characteristic urea absorption bands decreased in intensity, confirming their consumption during oxidation.

## Results and Discussion

After the successful mechanical recovery of the rim wires, they were consequently acid-treated as mentioned earlier to remove loosely bound metal oxide, following a heat treatment to remove residual organic contaminants. Then, the acid-treated rim wires were soaked in a solution of 3 M NaOH to promote uniform corrosion of the surface. To facilitate the corrosion, 1 mg of bleaching powder (Ca­(OCl)_2_) was added and stirred. After a quick stirring, the electrode was soaked in the same solution for 10 days continuously. During this corrosion-induced activation period, a HER LSV (Figure 1a) and an OER LSV (Figure 2a) were obtained at an interval of 24 h for 10 days.

**Figure 1:**
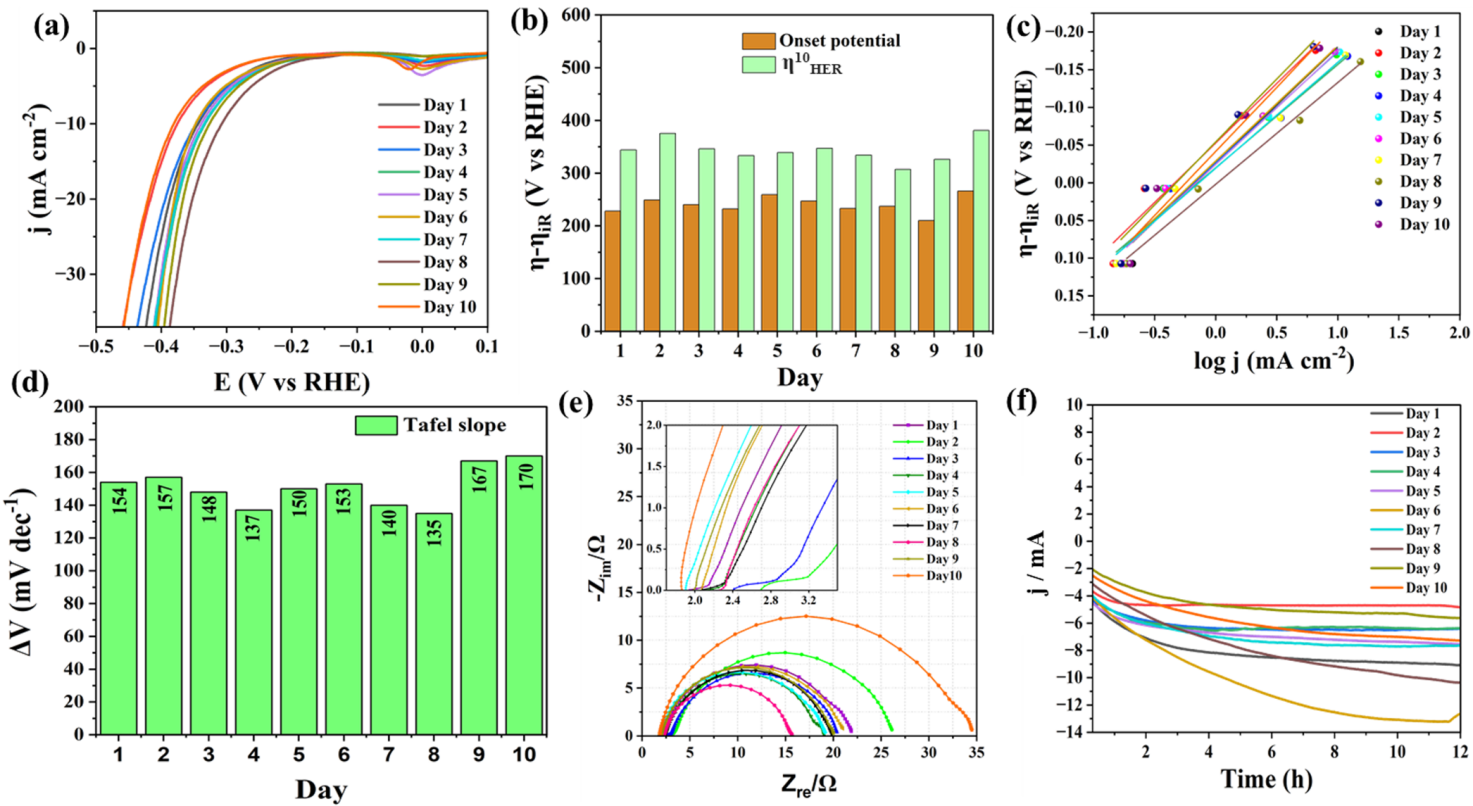
(a) HER LSVs acquired at regular intervals of corrosion of the recovered Fe-rich rim wire in 1.0 M KOH at 5 mV s^–1^. (b) Comparison of the onset overpotential and the overpotential at 10 mA cm^–2^ during the same. (c) Corresponding Tafel lines constructed from SCV responses. (d) The trend observed with Tafel slopes during the activation. (e) Corresponding Nyquist plots. (f) 12 h stability curves from CA tests.

**Figure 2:**
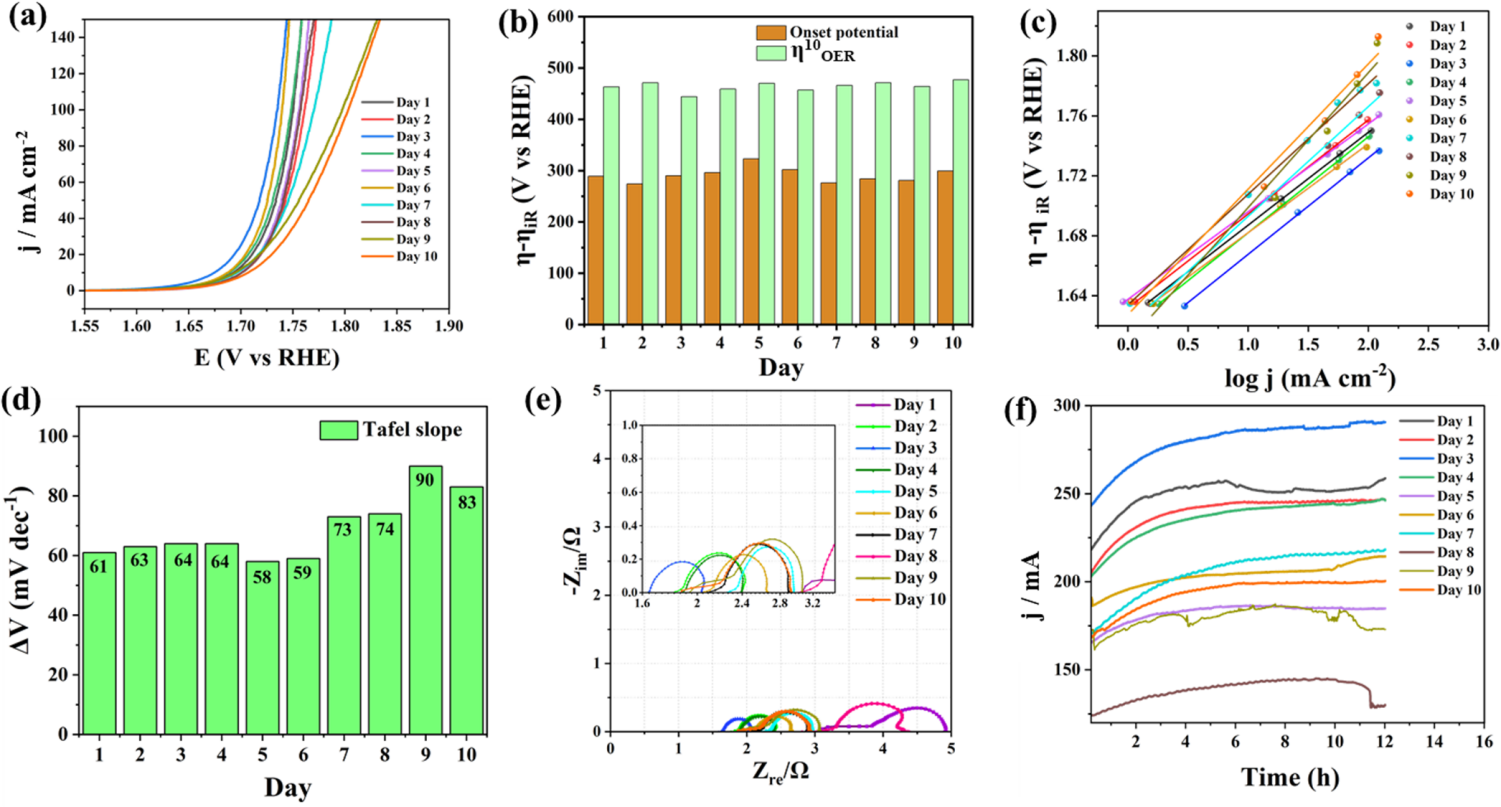
(a) OER LSVs acquired at regular intervals of corrosion of recovered Fe-rich rim wire in 1.0 M KOH at 5 mV s^–1^. (b) Comparison of the onset overpotential and the overpotential at 10 mA cm^–2^ during the same. (c) Corresponding Tafel lines constructed from SCV responses. (d) The trend observed with Tafel slopes during the activation. (e) Corresponding Nyquist plots. (f) 12 h-long stability curves from CA tests.

Using the recorded LSVs, the onset overpotential and the overpotential at 10 mA cm^–2^ were monitored and plotted as shown in Figures 1b and 2b for HER and OER, respectively. From these, it can be judged that the recovered rim wires obtained optimal characteristics for HER on the eighth day of corrosion-induced activation, and for OER, it was the third day. It clearly indicates that the surface modifications that occurred followed different trends for the HER and the OER. This is justifiable because the HER and the OER in alkaline solutions follow different mechanisms. For example, the kinetics of the OER in alkaline solutions depend largely on how quickly the OH* is adsorbed and oxidized to the O*. Following this, the O* can either take up another hydroxide to electrochemically form OOH* capable of dissociating into O_2_ or couple chemically with another O* to deliver O_2_.^5,49–53^ On the other hand, the kinetics of HER depends largely on the optimal adsorption free energy of H*.

However, Fe is inherently poor when it comes to HER, which explains why the onset overpotential is relatively higher than that of the state-of-the-art. However, in alkaline solutions, it is not just the adsorption free energies of H* but also the efficient and concurrent dissociation of the H–O bond in the water molecule, a relatively easier source to pick H^+^ for HER in a H^+^-scarce environment.^54,55^ The current understanding is that, in alkaline media where no free H^+^ ions are available, having a metal hydroxide cocatalyst is set to assist the catalyst by accelerating the dissociation of the H–O bond in the water molecule. Having these differences in their mechanisms, it is no wonder that the optimization periods are so different for OER and HER. In a similar fashion, the Tafel analysis was carried out for HER (Figure 1c) and OER (Figure 2c). Surprisingly, the lowest Tafel slopes for HER and OER were obtained on the eighth and fifth days of activation (Figures 1d and 2d, respectively), which do not match the activity trend discussed above. This implies that enhanced activity was not just a result of intrinsic activity changes, which usually change the Tafel slopes accordingly, but extrinsic factors like increased surface area could have acted together, too. Given the fact that the activation involves a 10-day-long corrosion, such changes in surface area are very likely to occur and overshadow intrinsic activity-directed Tafel slopes. To further fathom the observed trend, EIS analysis was also carried out along with the two other studies at the same time interval for the HER (Figure 1e) and (Figure 2e). As expected, the charge transfer resistance (*R*_ct_) was the lowest on the eighth day for HER and on the third day for the OER, which explains why better activities were observed for electrodes corroded for those durations. Interestingly, the uncompensated resistance (*R*_u_) that is supposed to be a constant in most of the electrochemical studies carried out under identical conditions kept changing with the corrosion, strongly hinting at significant surface changes. There was no specific trend in the changes seen with R_u_ values, implying it is not intrinsic but relies largely on the environment in which it is being corroded. After all this, the most intriguing observations were made when the stability of those electrodes was tested at the same set potentials in the catalytic turnover conditions of HER (Figure 1f) and OER (Figure 2f). All of the electrodes exhibited a significant enhancement in their activity in the first few hours of CA and were stable for the remainder of the 12 h CA test. This implies that the in situ electrochemical activation took place notably during the stability test, positively boosting the electrode’s performance in both HER and OER. The overall HER and OER studies were explicit in testifying how good a catalytic electrode the repurposed bike tire rim wire is.

Encouraged by these findings, we were motivated to explore the electrocatalytic properties of the same in the oxidation reactions of methanol, ethanol, and urea. Though it is evident that these 3d metal-based electrocatalysts are incapable of performing these oxidation reactions at potentials suitable for direct methanol/ethanol/urea fuel cells, they can still oxidize these molecules at much lower potentials than the potentials of the OER and can be used for wastewater valorization to make used carboxylates while producing H_2_ as a byproduct. The optimized OER electrode was screened in separate 1.0 M KOH solutions containing methanol, ethanol, and urea (0.5 M) to screen its MOR, EtOR, and UOR activities by using LSVs acquired at 5 mV s^–1^ (Figure 3a). Among them, UOR on the repurposed Fe-rich rim wire electrode occurred at a potential that is 200 mV lower than that of OER and MOR. When compared to EtOR, the same is 156 mV lower for UOR. Interestingly, only a slight cathodic shift was observed for MOR from OER. This suggests that the repurposed Fe-rich rim wire could be a better catalyst for UOR and slightly better for EtOR compared to MOR and OER. Hence, the repurposed electrode can be used for wastewater valorization that is rich in urea and ethanol. Besides, the observations made also reveal important information on the mechanisms of these reactions. In fact, methanol oxidation is used as a probe to find the adsorption fashion of OH on the electrode.

**Figure 3:**
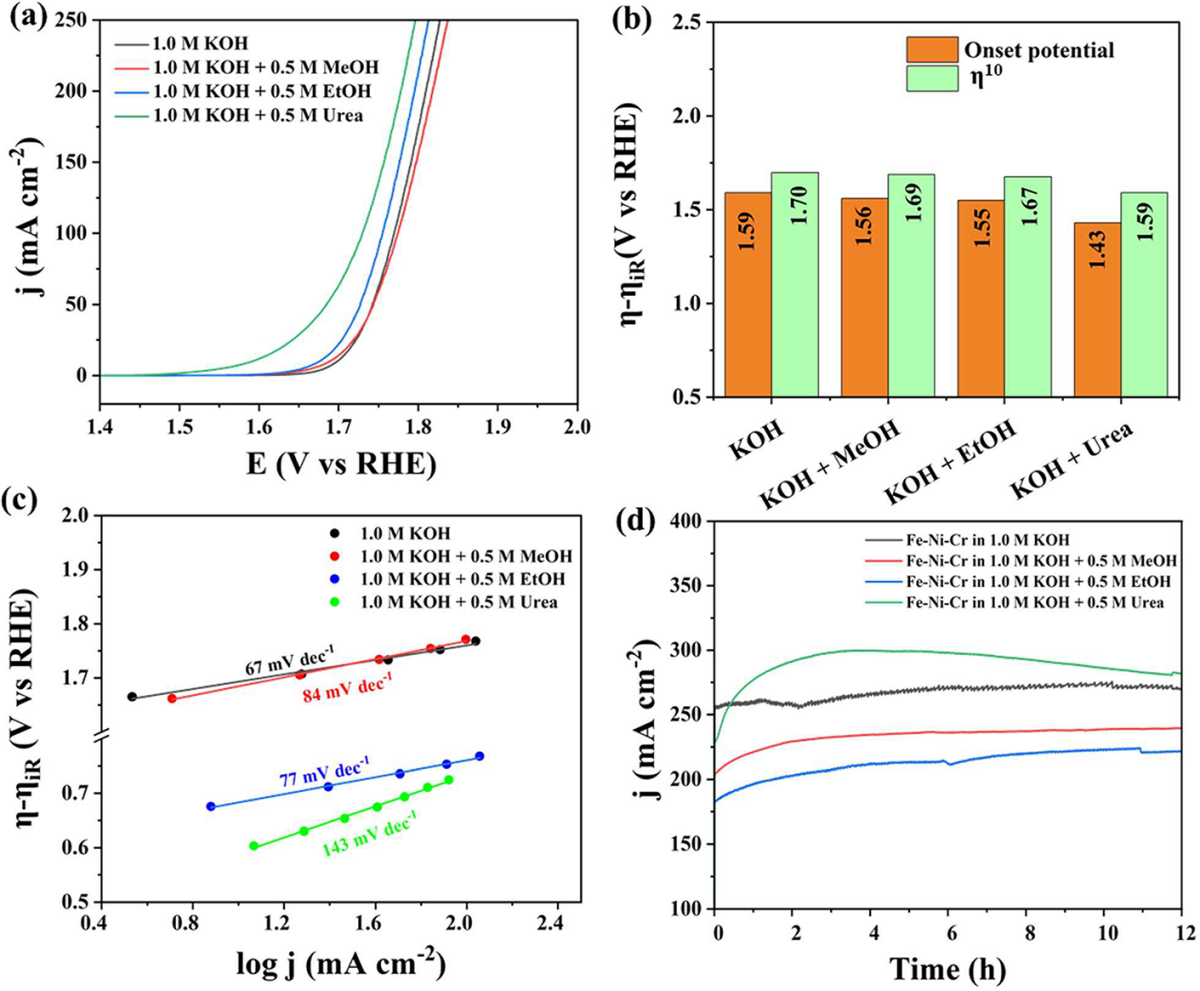
(a) LSVs for the OER (black), MOR (red), EtOR (blue), and UOR (green) acquired at 5 mV s^–1^. (b) Overpotentials (at onset and at 10 mA cm^–2^) comparison. (c) Corresponding Tafel lines. (d) 12 h-long stability curves (from CA) for the same.

If OH is bound strongly with the surface of the electrode, it increases the surface coverage of the same. Methanol oxidation proceeds swiftly when the OH is adsorbed strongly on the surface of the electrode.^56–59^ The fact that MOR and OER currents are very similar on the surface of the electrode indicates that the OH adsorption is not the rate-limiting step in the OER. It also suggests that the OER occurs primarily via the adsorbate evolution mechanism (AEM), and no oxygen in the lattice of the electrode material takes part in the mechanism.

A cathodic shift by 50 mV with EtOR indicates that ethanol oxidation is dependent on (solely) adsorbed OH on the surface of the electrode. It is anticipated because, in EtOR, the rate-limiting steps are the activation of −CH_2_–OH and CH_3_–CH_2_– bonds. Between C–O and C–C activations, the former do not rely largely on adsorbed OH but on −CH_2_–OH adsorption.^59^ This explains why EtOR is relatively better catalyzed than MOR on the repurposed Fe-rich rim electrode. However, C–C activation still needs strong OH adsorption for further oxidation to occur.^59^ Since the OH adsorption is apparently weak on the surface of the repurposed Fe-rich rim electrode, the oxidation of ethanol beyond acetic acid was not observed, as confirmed by postelectrolysis product analysis. With the UOR in place, the mechanism changes completely. The oxidation of urea (UOR) relies mostly on the preferential adsorption of NH_2_ groups on the electrode surface and does not need adsorbed OH. Hence, it required much lower potential to begin UOR compared to MOR and EtOR. As the electrode has been found to be adsorbing OH feebly, the oxidation of MOR and EtOR did not lead to any gas evolution, hinting that the oxidation was controlled with just four-electron and four-proton transfers forming corresponding carboxylic acids (formic acid from MOR and acetic acid from EtOR). The same was confirmed by analyzing the electrolyte after 1, 3, and 6 h of MOR (Figure S1a,b) and EtOR (Figure S2a,b) by using ^1^H and ^13^C NMR, respectively. The UOR to gaseous N_2_ and CO_2_ cannot be analyzed in similar ways. Hence, we used FTIR to prove the same. The FTIR spectra taken after 1, 3, and 6 h revealed a gradual reduction in the C=O stretching (Figure S3a,b), indicating the consumption of urea. Figure 3b shows the overpotential comparison of all four reactions studied, from which the superiority of UOR can be witnessed. The Tafel lines (Figure 3c) obtained from corresponding SCV curves revealed that though the UOR is better by apparent activity, its kinetics is too slow compared to that of the OER, which is attributed to the nonreliance of UOR on OH adsorption and weak NH_2_ (from urea) adsorption. The stability curves (Figure 3d) indicated stable performances for the OER, MOR, and EtOR, but for the UOR, the activity was enhanced initially and then stabilized. This could be due to ongoing surface reconstruction of the electrode in the presence of urea, a well-known structure-directing agent in material synthesis. A detailed EIS analysis (Figure 4a–d) carried out for all these reactions revealed that the *R*_ct_ is the lowest for UOR and follows the same order observed in Figure 3a. The *R*_u_ and *R*_ct_ are compared in Figure 4b for better viewing. The Bode phase angle plot (Figure 4c) showed that UOR exhibited the lowest relaxation time, resonating well with the activity and *R*_ct_ trends as expected. The Bode absolute impedance plot (Figure 4d) revealed the highest admittance for UOR at the lowest frequency, implying better charge transfer with UOR. The overall electrochemical studies revealed that the OER occurs via AEM, as the repurposed Fe-rich rim wire electrode did not adsorb OH strongly. Electrochemical activity between the OER and other electrolytes, MOR, EtOR, and UOR is compared in Table 1.

**Figure 4:**
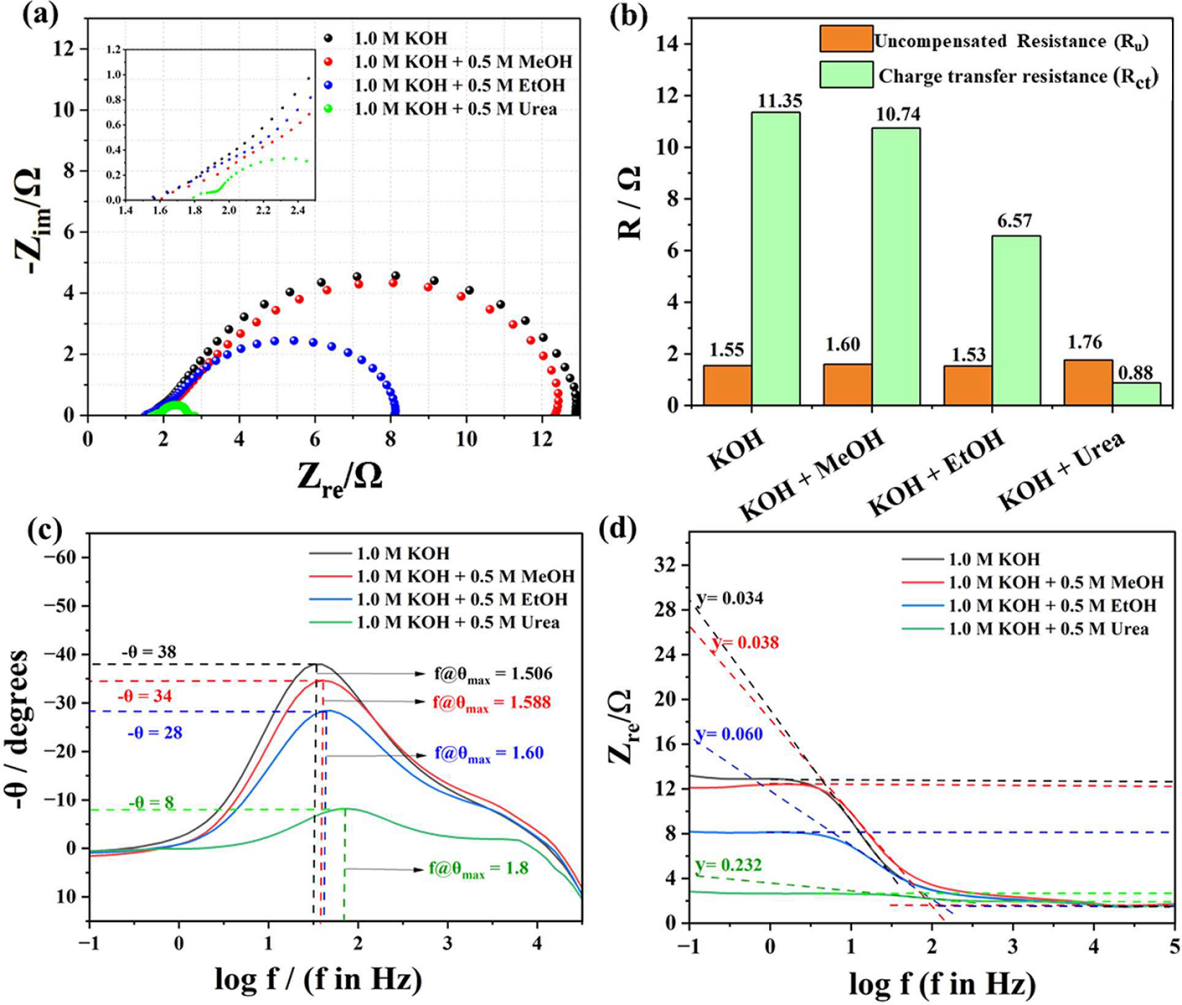
(a) Nyquist plots of the OER, MOR, EtOR, and UOR acquired at 1.624 V vs RHE. (b) Comparison of their corresponding *R*_u_ and *R*_ct_. (c,d) Respective Bode phase angle and Bode absolute impedance plots.

**Table 1:** Electrochemical Activity Comparison of OER with MOR, EtOR, and UOR^a^

electrolyte	onset potential (V)	overpotential @10 mA/cm^–2^ (V)	Tafel slope (mV/dec)	*R*_s_	*R*_ct_
1.0 M KOH	1.59 (360 mV)	1.70 (470 mV)	67	1.81	10.87
1.0 M KOH + 0.5 MeOH	1.56	1.69	84	2.04	10.27
1.0 M KOH + 0.5 EtOH	1.55	1.67	77	2.20	5.92
1.0 M KOH + 0.5 Urea	1.43	1.59	143	1.94	0.70

a Difference between theoretical and experimental value for OER given in parentheses (*E*_exp_– *E*_1.23 V_).

Moreover, the weak OH adsorption was responsible for the controlled oxidation of methanol and ethanol to formic acid and acetic acid. These insights can be extended to controlled oxidation of primary alcohols and to produce H_2_ through water-urea coelectrolysis.

Following this, to understand the surface properties (physical and chemical) and correlate them with the observed activity trends above, the recovered Fe-rich rim wire was extensively characterized by several techniques, as discussed below. Figure 5a shows the XRD pattern of the recovered Fe-rich rim wire electrode. The observed pattern could be matched with the reference patterns of Fe with powder diffraction file (PDF) numbers 06-0696, FeNi (PDF: 37-0474), FeCr (PDF: 34-0396), and FeCrNi (PDF: 35-1375). However, the best match was found with the latest, indicating that the rim wire could be made of an Fe-rich stainless-steel alloy. Following this, SEM images with gradually decreasing magnifications were obtained (Figure 5b–e), which revealed a uniform ribbon-like structure that appears to have been laid partially on top of one another like sticky notes on a notice board. Using the ED spectrum, the elemental composition (Figure 5f) of the same was analyzed. Surprisingly, the composition analysis revealed the exclusive presence of Fe by 99.76% with trace levels of Cr and Ni. Between Cr and Ni, Cr was found to be more than five times of what Ni was. This is almost a pure Fe wire with trace Cr and Ni dopants. The elemental EDS mapping (Figure 5g–i) proved the same, and the distribution of these elements was found to be uniformly spread over the region chosen for the analysis. The corresponding ED spectrum showed no other elements (Figure 5j). Since the surface structures seen could not be segregated by the standard ultrasonication procedure, transmission electron microscopy (TEM) analysis could not be done. The chemical state of almost omnipresent Fe, along with that of O and C, was analyzed using XPS survey and narrow scans of Fe 2p, O 1s, and C 1s states (Figure 6a–d). The survey scan exhibited peaks that are only characteristic of Fe, Cr, Ni, O, C, Na, and Ca, and no other impurities were detected. While Fe, Cr, Ni, O, and C could be inherent to the recovered rim wire, Cl, Na, and Ca should have been from the NaOH and Ca­(OCl)_2_ used to corrode the wire. The Fe 2p scan (Figure 6b) showed the presence of both the Fe^2+^ and Fe^3+^ states, as expected.

**Figure 5:**
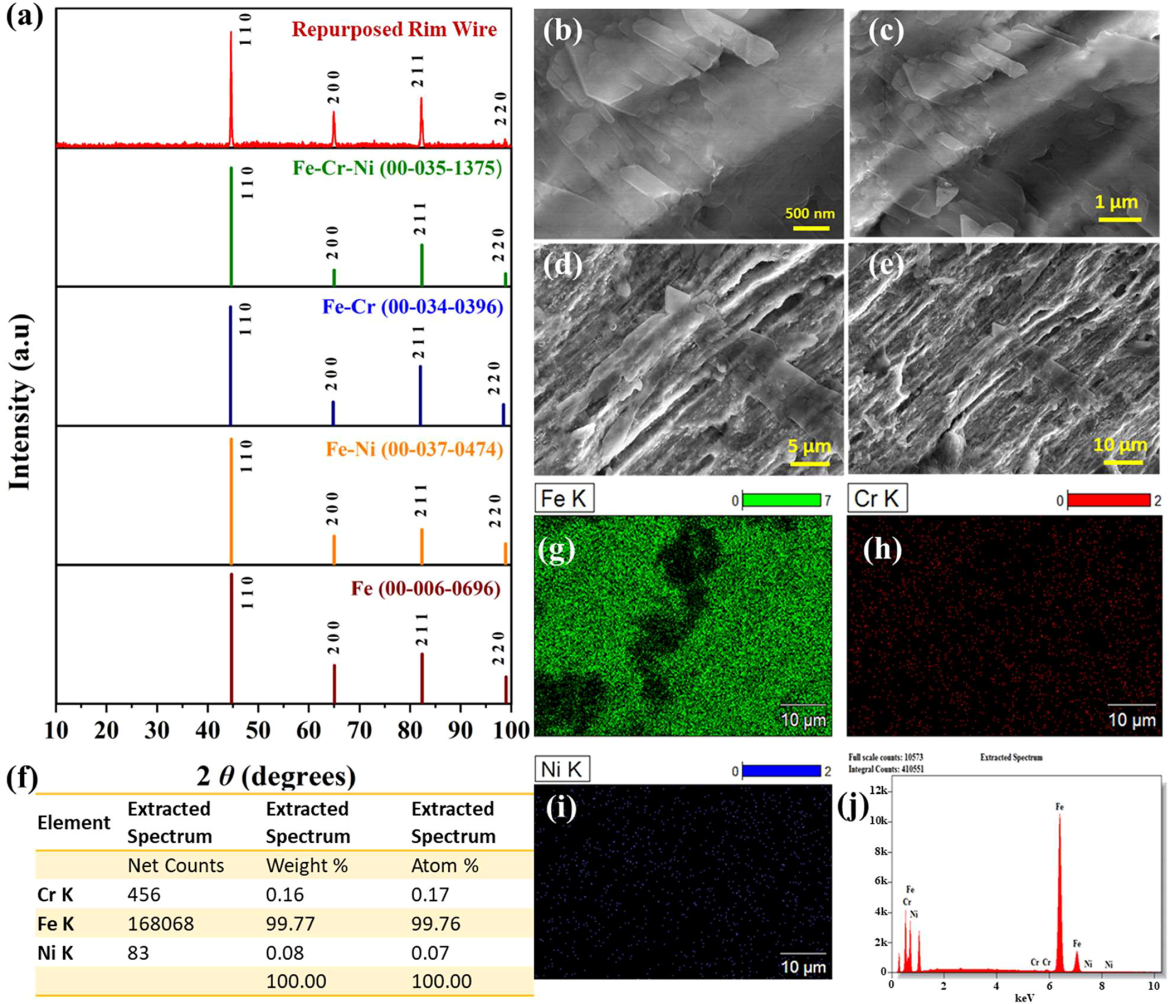
(a) XRD pattern of recovered Fe-rich rim wire compared with the standard reference patterns. (b–e) FE-SEM images of the same with lower magnification. (f) Elemental composition table. (g–i) Corresponding EDS elemental mapping of Fe, Cr, and Ni, respectively. (j) ED spectrum of the same showing no other impurities.

**Figure 6:**
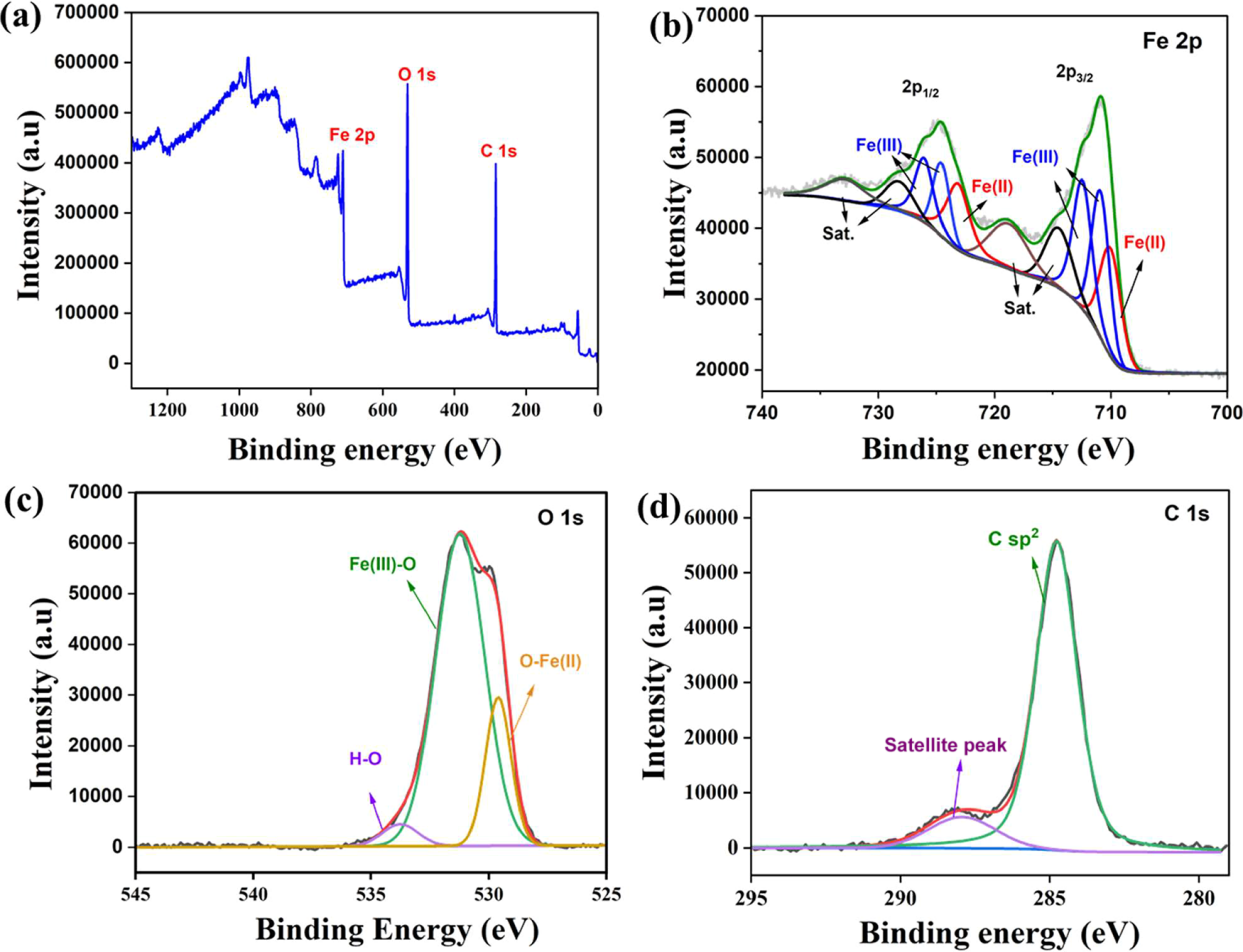
(a) XPS wide scan of recovered Fe-rich rim wire from discarded bikes. (b–d) Corresponding narrow scans of Fe 2p, O 1s, and C 1s, respectively.

The O 1s scan (Figure 6c), on the other hand, showed the same alongside an O–H moiety peak on a slightly higher binding energy side, confirming the observations made with the Fe 2p scan. The C 1s (Figure 6d), featuring mainly the sp^2^ carbon, was used as the reference for calibration of all other peaks. The observations made in XPS analysis matched closely with other similar studies reported in the literature.^36–38^ From this, it can now be claimed that the moderate HER and OER activity was mainly due to the fact that the recovered rim wire is primarily just Fe (up to 99.76%), and the carboxylic acid selectivity observed in MOR and EtOR is mainly due to the poor OH adsorption witnessed with electrodes that are made exclusively with Fe. In addition, the appreciable UOR activity might be due to the optimal Fe–N interaction energies, unlike the weaker Fe–O interactions.

To find out the surface changes and stability of the material, post-stability analysis, such as SEM and XPS, was conducted, and EDX with elemental mapping for both after HER and OER was performed. In SEM-EDS analysis carried out forthe electrode after HER studies, the surface of the material did not change that much (Figure S4a-j) and upon applying negative potential some of the oxidized irons should be reduced but due to the formation of productive oxide layer, we did not get a strong metallic iron signal. When screened with XPS, only a low intense Fe peak could be seen (Figure S5a-d). After OER analysis, the surface changed due to formation of Fe_2_O_3_, which was present as (FeO_4_)^2–^ at high potential during the activation of the material (by running 50 cycles of CV). So, after OER analysis, EDX mapping shows an oxygen-rich surface (Figure S6a-j) and at the same time XPS analysis shows the abundant presence of the element Fe^2+^ due to the formation of Fe_2_O_3_ during the activation (Figure S7a-d). The catalytic OER performance of our catalyst compared with the already-reported iron oxide-based material in the literature is given in Table 2.

**Table 2:** OER Performance Comparison with the Literature

catalyst	pH	overpotential (mV) @10 mA cm^–2^ (OER)	Tafel slope (mV/dec)	ref no.
Fe_3_O_4_	14	470	67	this study
Fe_2_O_3_-A	14	460	106	^60^
Fe_2_O_3_-B	14	480	107	
Fe_3_O_4_@NiO/graphene/C_3_N_3_	14	288	40.46	^61^
Fe_3_O_4_ NPs	14	300		^62^
Fe_3_O_4_ NPs	14	234	100	^63^
Co–Fe_3_O_4_ NPs	14	210		^64^
Ni–Co–Fe_3_O_4_	14	234	54.84	^65^
SMNF-Fe3O_4_	14	217.3	89.4	^66^
Ni–Fe_3_O_4_(001)	14	340(@5 mA cm^–2^)	57	^67^
Fe_3_O_4_–Vac	14	353	50	^68^
FeOOH	14	853	170	
Fe_3_O_4_	14	415	53	
Fe_3_O_4_/Ni-BDC	14	295	47.8	^69^
Ni/NiO/Fe_3_O_4_	14	288	62	^70^

## Conclusions

This study demonstrates a sustainable and cost-effective approach to repurpose Fe-rich bike rim wires into functional electrocatalysts for the HER, OER, and small-molecule oxidation reactions. Through a simple corrosion-induced surface activation in NaOH and Ca­(OCl)_2_ solution, the recovered wires showed distinct optimal activation times for HER and OER (8 and 3 days, respectively), highlighting their mechanistic differences in alkaline media. Electrochemical analyses, including LSV, Tafel, EIS, and CA, confirmed that both intrinsic activity and extrinsic surface modifications contributed to the observed performance. The repurposed electrode showed stable HER and OER activity with signs of in situ activation under operational conditions. Notably, the same electrode demonstrated promising activity in the UOR, EtOR, and MOR, with UOR requiring the lowest onset potential and exhibiting the most favorable charge-transfer characteristics. Mechanistic insights obtained from product analyses and FTIR spectroscopy suggest that the weak OH adsorption on the Fe surface led to selective oxidation of alcohols to carboxylic acids without further degradation, while the superior UOR activity was likely facilitated by Fe–N interactions rather than OH adsorption. Compositional and surface analyses confirmed the wire to be predominantly Fe (99.76%) with trace Cr and Ni, supporting the correlation between structure and function. Overall, this work not only adds value to metallic waste but also offers mechanistic insights for guiding future low-cost catalyst design for sustainable hydrogen production and wastewater valorization.

## Supplementary Material



## Data Availability

All the data related to this study are included in the main article and the supporting data in the Supporting Information.

## Notes

Figure S1, product confirmation of MOR; Figure S2, product confirmation of EtOR; Figure S3, proving the consumption of urea with time during UOR using FT-IR spectroscopy; Figure S4, FE-SEM images of after HER analysis with loweringmagnification, elemental composition table, corresponding EDSelemental mapping of Fe, Cr, Ni, and O, ED spectrum ofthe catalyst after HER, and elemental composition table; Figure S5, XPSwide scan of recovered Fe-rich rim wire after HER stability, and correspondingnarrow scans of Fe 2p, O 1s, and C 1s; Figure S6, FE-SEMimages of after OER analysis with lowering magnification, elementalcomposition table, corresponding EDS elemental mapping of Fe, Cr, Ni, and O, ED spectrum of the catalyst after OER, and elemental composition table; Figure S7, XPS wide scan of recovered Fe-richrim wire after OER stability and corresponding narrow scans of Fe 2p, O 1s,and C 1s (PDF)
