# Supplementary material for: Turning Fe-Rich Waste into an Advanced Electrocatalyst for the Production of H2 and Useful Carboxylates
Source: ACS Omega. 2025 Dec 8;10(50):61841–51. doi: 10.1021/acsomega.5c08418 (PMC12750195; doi:10.1021/acsomega.5c08418)
Supplement: Supplementary file 1 [file ao5c08418_si_001.pdf]

Supporting Information

Turning Fe-rich Waste into An Advanced Electrocatalyst for the Production H_2 and Useful Carboxylates

Karthik Eswaran,[a] Rajini Murugesan,[a] Arthanareeswari Maruthapillai,[a] and Anantharaj Sengeni*[b]*

a. *Department of Chemistry, SRM Institute of Science and Technology, Kattankulathur 603203, Tamil Nadu, India.*

b. *Laboratory for Electrocatalysis and Energy, Department of Chemistry, Indian Institute of Technology, Kanpur 208 016, Uttar Pradesh, India.*

† Correspondence should be addressed to arthanam@srmist.edu.in and ananths@iitk.ac.in.

Summary of Content

S. No.	Item	Quantity and Pages
**1**	Pages	8 (S1-S9)
**2**	Additional Experimental Details	S2
**3**	Figures	7 (S2-S8)
**4**	Tables	0

Materials and chemicals

Bire tire was got collected from nearby local shop, Sodium hydroxide(NaOH), Potassium hydroxide (KOH), Urea (H2NCONH2), leishman stain solution (CH3OH) were purchased from SRL chemicals, commercially available bleaching power was purchased from nearby local shop and ethanol (CH3CH2OH) was purchased from Chanshu Hongsheng Chemical Co Ltd.

Product Confirmation for MOH oxidation

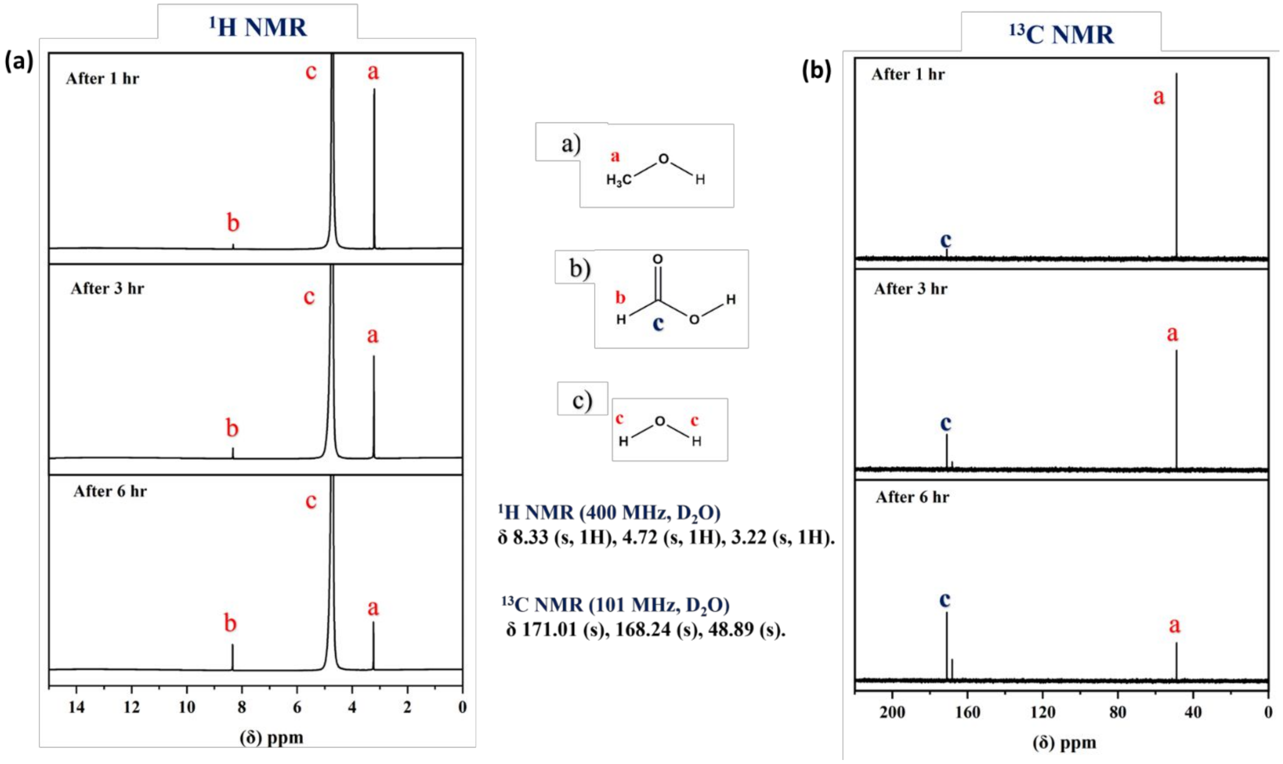

Figure S1: Product confirmation of MOR (a) ^{1}H and and (b)^{13}C NMR.

Product Confirmation for EtOH oxidation

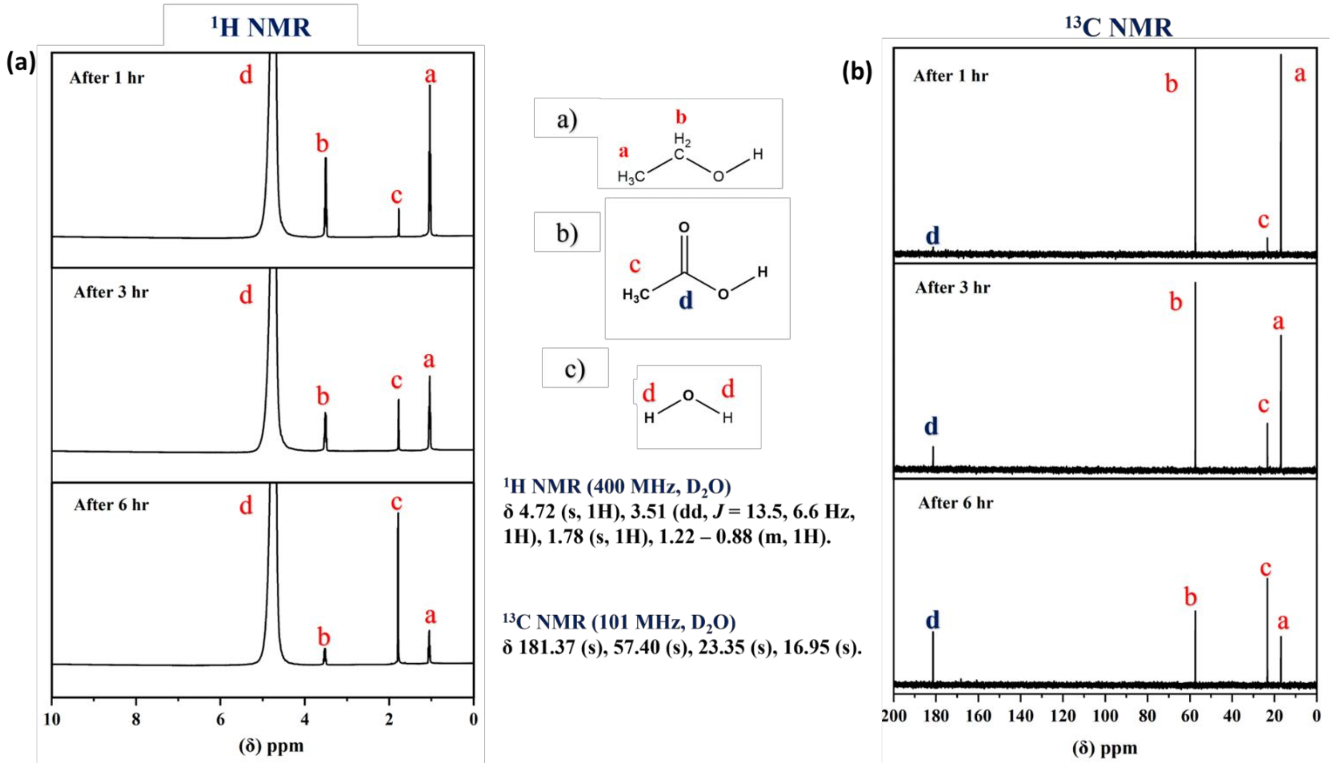

Figure S2: Product confirmation of EtOR (a) ^{1}H and (b)^{13}C NMR.

Product Confirmation for UOH oxidation

a)

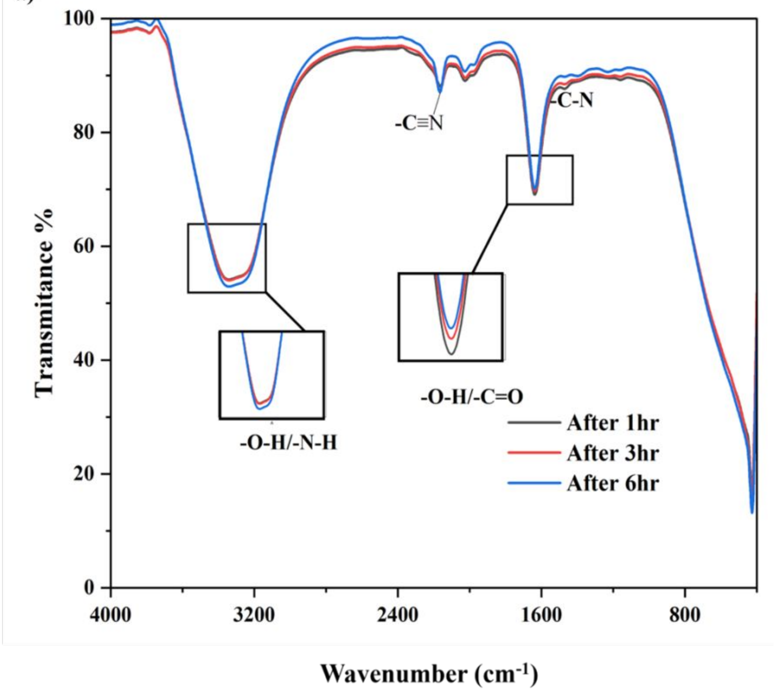

b) spectrum result for urea oxidation with ıe period

Molecular bond	Value (cm^{-1})
C-N (Stretching)	1463
-O-H (Bending)<br>-C=O (Stretching)	1631
-C≡N (Stretching)	2166
-OH/-N-H (Stretching)	3351

Note :

$H_2NCONH_2 + KOH \rightarrow KOCN + NH_3 + H_2O$

Once we add Urea inside bulk KOH that time KOCN also formed it show -C≡N (Stretching) At 2166 cm^{-1}

Figure S3: Proving the consumption of urea with time during UOR using FT-IR spectroscopy (a) for which the corresponding stretching frequencies can be found listed in (b).

Post stability SEM analysis for HER

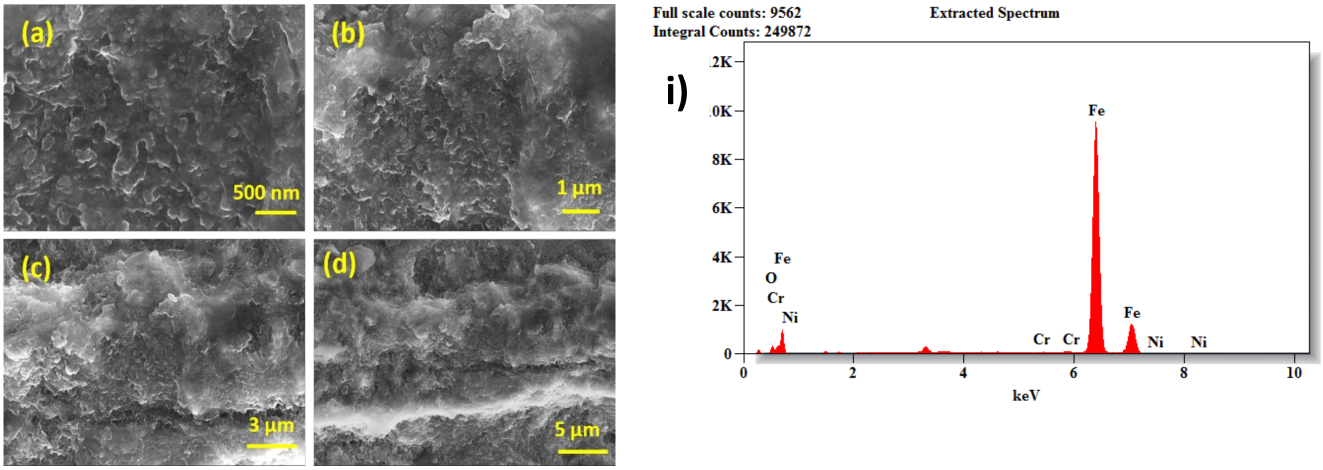

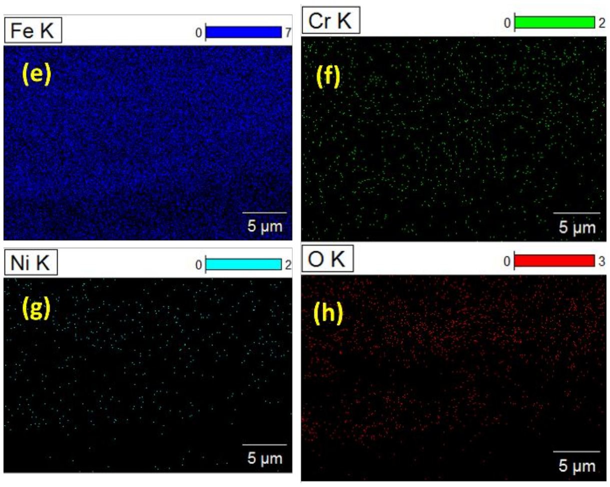

j)

Element	Extracted Spectrum	Extracted Spectrum	Extracted Spectrum
	Net Counts	Weight %	Atom %
**O K**	61	0.12	0.43
**Cr K**	311	0.12	0.13
**Fe K**	155945	99.61	99.31
**Ni K**	136	0.14	0.13
		100.00	100.00

Figure S4: (a-d) FE-SEM images of after HER analysis with lowering magnification. (f) Elemental composition table. (e-h) Corresponding EDS elemental mapping of Fe, Cr and Ni and O respectively. (i) The ED spectrum of the catalyst after HER and (j) Elemental composition table.

Post stability XPS analysis for HER

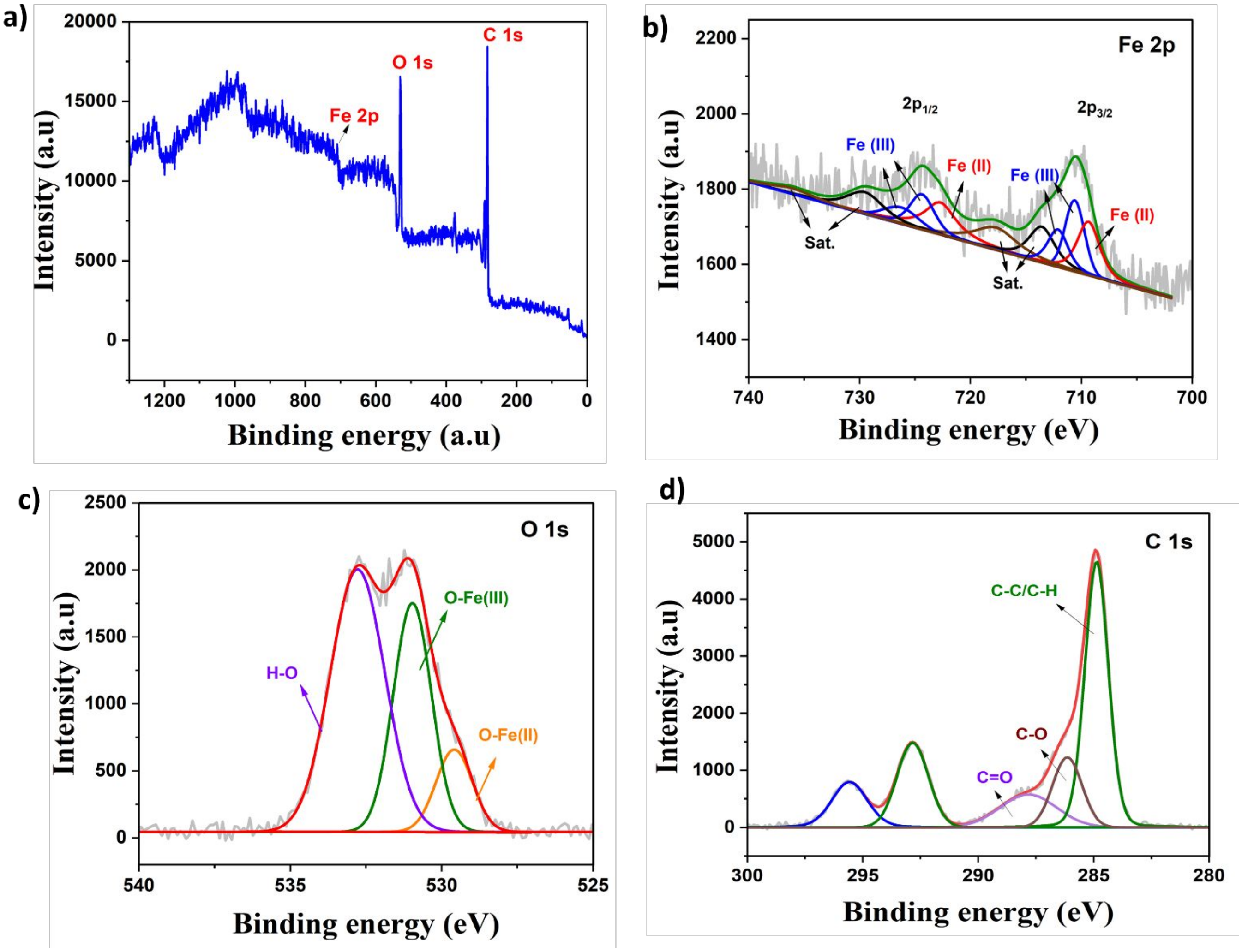

Figure S5: (a) XPS wide scan of recovered Fe-rich rim wire after HER stability. (b-d) Corresponding narrow scans of Fe 2p, O 1s, and C 1s, respectively.

Post stability SEM analysis for OER

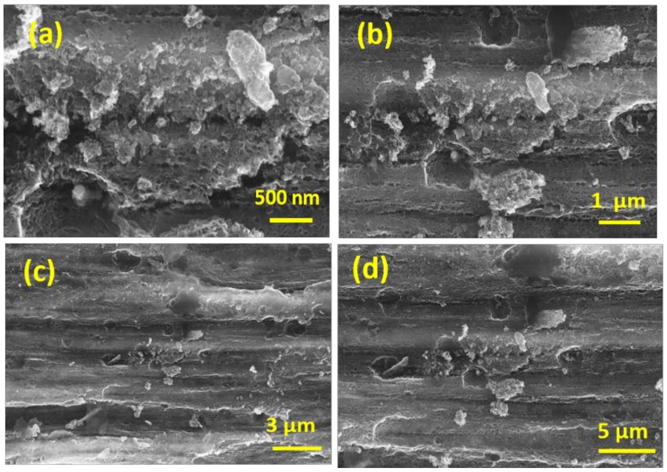

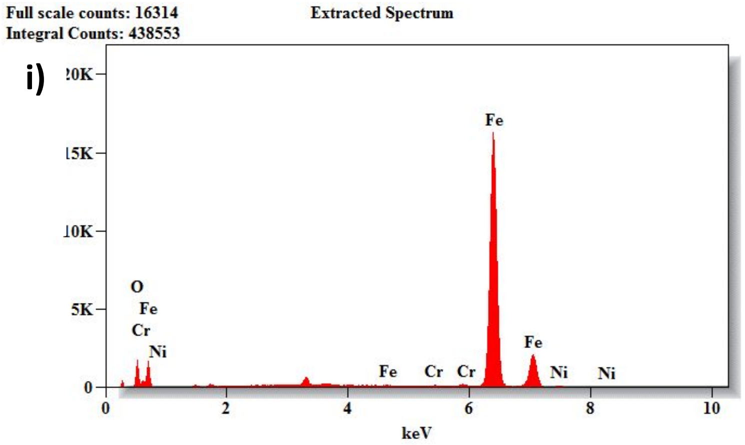

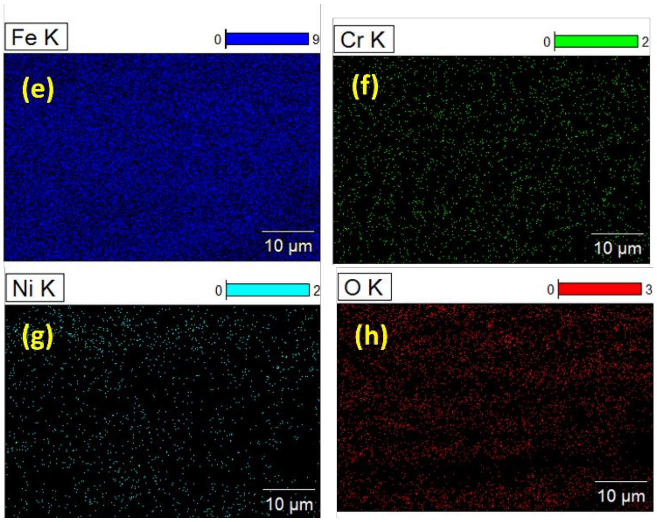

j)

Element	Extracted Spectrum	Extracted Spectrum	Extracted Spectrum
	Net Counts	Weight %	Atom %
**O K**	6213	6.75	20.17
**Cr K**	411	0.10	0.09
**Fe K**	258194	92.40	79.12
**Ni K**	1332	0.75	0.61
		100.00	100.00

Figure S6: (a-d) FE-SEM images of after OER analysis with lowering magnification. (f) Elemental composition table. (e-h) Corresponding EDS elemental mapping of Fe, Cr and Ni and O respectively. (i) The ED spectrum of the catalyst after OER and (j) Elemental composition table.

Post stability SEM analysis for OER

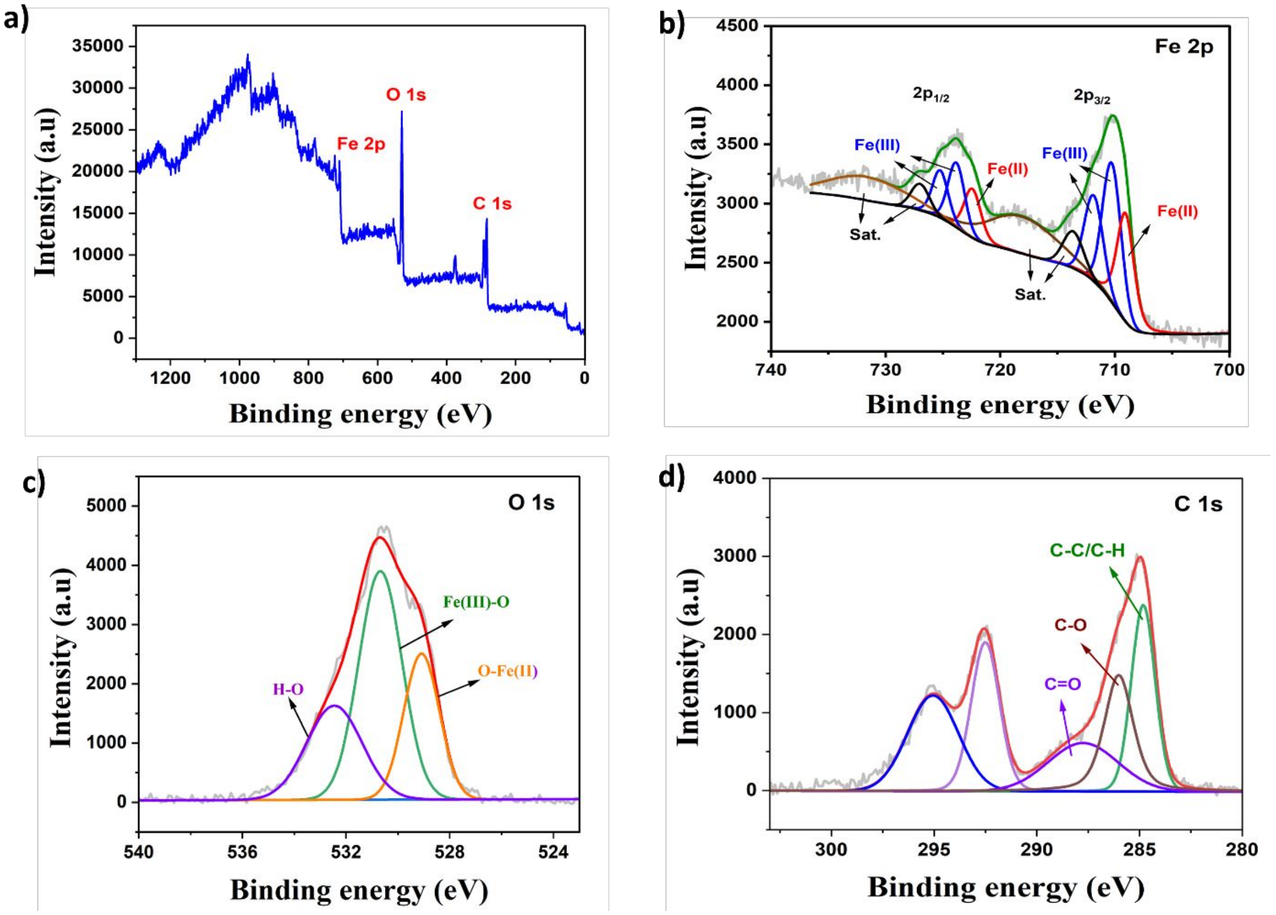

Figure S7: (a) XPS wide scan of recovered Fe-rich rim wire after OER stability. (b-d) Corresponding narrow scans of Fe 2p, O 1s, and C 1s, respectively.
